# Synaptic Plasticity and Quantized Conductance States in TiN-Nanoparticles-Based Memristor for Neuromorphic System

**DOI:** 10.1186/s11671-022-03696-2

**Published:** 2022-06-10

**Authors:** Chandreswar Mahata, Muhammad Ismail, Myounggon Kang, Sungjun Kim

**Affiliations:** 1 Division of Electronics and Electrical Engineering, Dongguk University, Seoul, 04620 Republic of Korea; 2 Department of Electronics Engineering, Korea National University of Transportation, Chungju-si, 27469 Republic of Korea

**Keywords:** Resistive switching, Al-doped HfO_2_, ALD TiN-nanoparticles, Quantum conductance, Synaptic plasticity

## Abstract

Controlled conductive filament formation in the resistive random access memory device is an essential requirement for analog resistive switching to develop artificial synapses. In this work, we have studied Au/Ti/HfAlO_x_/TiN-NP/HfAlO_x_/ITO RRAM device to demonstrate conductance quantization behavior to achieve the high-density memory application. Stepwise change in conductance under DC and pulse voltage confirms the quantized conductance states with integer and half-integer multiples of G_0_. Reactive TiN-NPs inside the switching layer helps to form and rupture the atomic scale conductive filaments due to enhancing the local electric field inside. Bipolar resistive switching characteristics at low SET/RESET voltage were obtained with memory window > 10 and stable endurance of 10^3^ cycles. Short-term and long-term plasticities are successfully demonstrated by modulating the pre-spike number, magnitude, and frequency. The quantized conductance behavior with promising synaptic properties obtained in the experiments suggests HfAlO_x_/TiN-NP/HfAlO_x_ switching layer is suitable for multilevel high-density storage RRAM devices.

**Supplementary Information:**

The online version contains supplementary material available at 10.1186/s11671-022-03696-2.

## Introduction

Resistive random access memory (RRAM) has become attractive and it shows promise for future non-volatile memory devices due to its simple structure of switching layer sandwiched between two electrodes, with high-density memory structure, high speed, and low electrical power consumption [1–3]. The principle working mechanism of RRAM device is believed to be due to the formation and dissociation of the conductive filaments (CFs) formed inside the switching layers by applying external electric fields [4, 5]. However, the stochastic nature of CFs formation during device operation can hinder the large-scale commercial application of the RRAM device. So several process optimizations such as doped switching layers, metal electrode selection, bilayer switching layer structures, and metal nanocrystal incorporation are needed to control the CFs formation to improve the device-to-device and cycle-to-cycle variability [6–13]. Among different techniques, nanoparticles embedded oxide-based switching layers are examined extensively, influencing CFs formation and improving the resistive switching process. Recently, Liu et al. have described that Cu NPs inside SiO_2_ can control the forming process and, by stabilizing the resistive switching, improve the endurance characteristics [14]. Ag embedded Al_2_O_3_:Ag:ZnO switching layer also exhibited a high I_on_/I_off_ ratio with gradual SET and RESET characteristics [15]. Ag-NPs included Al_2_O_3_ switching layers also show DC cycling stability due to enhancing the local electric field due to the presence of oxygen vacancies and Ag ions [16]. Bousoulas et al. described that multilevel memory state, better variability, and long retention were achieved by Pt-nanocrystals (NCs) inclusion inside TiO_x_ due to local field enhancement [10]. Also, embedding Pt-NCs inside switching layers, the local electric field leads to narrow and controlled CFs formation, further enhancing the switching performances described by Sakellaropoulos et al. [9]. In RRAM, gradual synaptic weight change manipulation by applying external stimuli is essential for high-density memory storage and artificial synaptic device for neuromorphic application. The presence of low oxygen vacancies that promotes gradual change in conductance states of nanoscale filamentary synaptic devices was observed [17, 18]. The atomic point contacts (APCs) were recently studied, which control the construction of the quantized conductance states. Change in quantized conductance states is the intrinsic property of the nanoscale CFs and is commonly found in oxide-based switching layers mediated by the formation and movement of oxygen vacancies and oxygen ions [4, 19, 20]. Stepwise increase in conductance due to precise atomic control of electron transport in conductive filaments leads to a higher memory density. *G*_*0*_ = 2*e*^*2*^*/h* (77 µS) is the quantum conductance unite, where *e* is the electronic charge, and the *h* is the Plank constant [21, 22]. The atomic layer deposition (ALD) technique is promising for achieving high-density metal nanoparticles with controlled thickness [23]. In this work, we have included ALD-based TiN-NP into HfAlO_x_ matrix to control the quantized conductance state and have studied the synaptic properties for neuromorphic applications. Finally, we have studied gradual conductance change with integer and half-integer multiples of G_0_ in both SET and RESET processes in detail for the RRAM device consisting HfAlO_x_/TiN-NP/HfAlO_x_ switching layer. Also, the short-term plasticity (STP) and long-term potentiation (LTP) were studied by controlling the pulse number and spike frequency.

## Experiments

Initially, commercially available indium tin oxide (ITO) (resistivity ~ 60 Ω/sq)-coated glass was used for device fabrication. The surface of ITO was washed stepwise with acetone, isopropyl alcohol, and DI water. After cleaning immediately, ITO samples were moved for the atomic layer deposition chamber (Lucida D100 thermal ALD). Nearly 5 nm of HfAlO_x_ (TMA 1 + TEMAH 2) was deposited before TiN deposition. HfAlO_x_ alloy ALD deposition details have been given in earlier work [24]. After deposition of HfAlO_x_ alloy layer, 20 cycles of TiN were deposited with a showerhead plasma-type ALD by titanium tetrachloride (TiCl_4_) and NH_3_ as precursors. Another layer of 5 nm HfAlOx alloy dielectric was grown to sandwich the TiN-NP inside HfAlO_x_ layers. Using a photolithography, Au/Ti bilayer top electrodes (TEs) were deposited by an e-beam evaporator with a deposition rate of 0.3 Å/s. A liftoff process in acetone achieved the top electrode with an area of 100 × 100 µm. Cross section analysis, elemental profile, the surface morphology of different deposited layers were scanned by field emission transmission electron microscope (JEOL JEM-F200) and field emission scanning electron microscope (JEOL-7800F). Keithley 4200 SCS semiconductor parameter analyzer along with a 4225-PMU ultrafast current–voltage (I–V) pulse module was utilized for all DC and pulse leakage current vs voltage (I-V) characteristics. All resistive switching properties were measured with the external electrical DC/pulse bias given to the top Au/Ti electrode, and the bottom electrode was in the ground.

## Results and Discussion

**Fig. 1:**
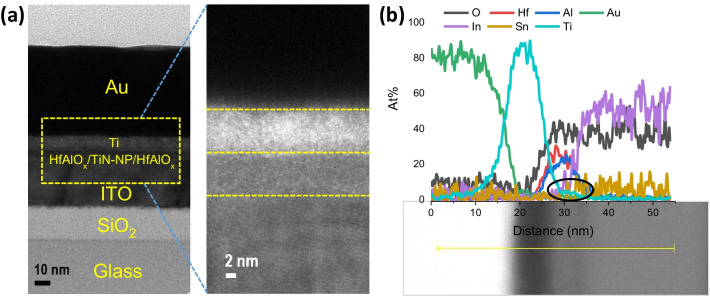
**a **Cross-sectional HRTEM of the Au/Ti/HfAlO_x_/TiN-NP/HfAlO_x_/ITO RRAM device. Right side magnified switching layer presented. **b** EDS line profile of the RRAM device measured from Au/Ti top electrode to ITO bottom electrode

After the sample preparation with a focused ion beam (FIB), the cross section of the Au/Ti/HfAlO_x_/TiN-NP/HfAlO_x_/ITO RRAM device was examined by the transmission electron microscope (TEM) as shown in Fig. 1a. From the TEM image, all the layers from the device are clearly shown. The thickness of the overall switching layer was ~ 10 nm which was similar to the target thickness controlled by the ALD recipe of AlO, HfO, and TiN cycles. Also, the cross-sectional energy-dispersive spectroscopy (EDS) analysis (Fig. 1b) was done to detect different layers of the device. The presence of Au, Ti, Al, Hf, O, and Sn was confirmed by the EDS line scan as shown in Fig. 1b. From the atomic percentage of Ti profile, the small shoulder of Ti intensity inside the HfAlO_x_ region was detected, which is believed to be emerged due to the presence of TiN-NP as highlighted by the black circles in Fig. 1b. Scanning transmission electron microscope (STEM) along with energy-dispersive spectroscopy (EDS) mapping is provided in Additional file 1: Fig. S1 to confirm all layers in the NP-based RRAM device. Although from the cross section TEM analysis, the presence of TiN-NP was not clear, which may be due to the similar color contrast of HfAlO_x_ and TiN-NP or the surface oxidation of the TiN surface during 2^nd^ layer of HfAlO_x_ deposition. Although due to higher atomic concentration of Ti from Au/Ti top electrode is dominated, the Ti intensity due to the presence of TiN-NP inside the HfAlO_x_ matrix was suppressed. In the previous work, the Ti presence was clearly demonstrated by the cross-sectional STEM image, core-level XPS spectra of TiN-NP, and elemental profile of Ti across the cross section of ITO/HfAlO_x_/TiN-NP/HfAlO_x_/ITO RRAM device [24]. In the supporting information, to observe the distribution of TiN-NPs surface morphology on the TiN-NP/HfAlO_x_/ITO structure, scanning electron microscope (SEM) image is presented in Additional file 1: Fig. S2. From Additional file 1: Fig. S2, a clear distribution of TiN-NP was achieved at a deposition temperature of 300 °C with 20 cycles of TiN ALD. High-density TiN-NPs distribution on HfAlO_x_ can be promising for RRAM applications for controlling oxygen vacancy and the formation of controlled conductive filaments during device switching operation. Variation in diameter of the NPs was observed (ranges from ~ 2 to 25 nm), which can be controlled by proper thermal treatment.

**Fig. 2:**
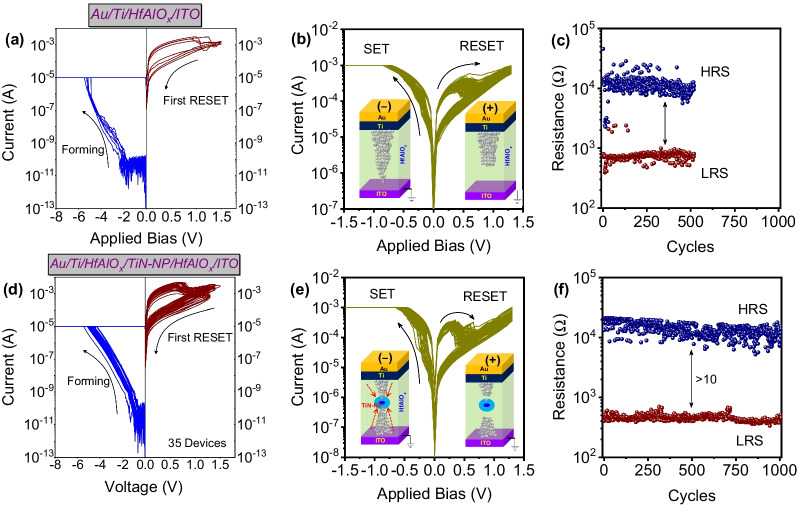
**a**, **d** Electroforming at 10 µA I_cc_ and 1^st^ RESET characteristics of multiple Au/Ti/HfAlO_x_/ITO and Au/Ti/HfAlO_x_/TiN-NP/HfAlO_x_/ITO RRAM devices. **b**, **e** Bipolar resistive switching properties with SET at 1 mA I_cc_ for both devices. Inset shows the schematics of switching mechanism during SET/RESET operations. **c**, **f** Endurance behavior of both Au/Ti/HfAlO_x_/ITO and Au/Ti/HfAlO_x_/TiN-NP/HfAlO_x_/ITO RRAM devices with 500 and 1000 DC cycles, respectively.

Figure 2a and d shows the electroforming and first RESET characteristics of Au/Ti/HfAlO_x_/ITO RRAM device with and without TiN-NP inclusion. Devices show forming voltages at the range of ‒4.6 V ~ ‒5.5 V as shown in the comparison characteristics in Additional file 1: Fig. S3. However, a reduction in the effective electric field during the forming process was observed after introducing TiN-NP inside HfAlO_x_ matrix as shown in Additional file 1: Fig. S3. However, the conduction mechanism before forming processes was changed, due to the presence of additional intrinsic defects after introducing TiN-NP as confirmed by the Additional file 1: Fig. S3. The uniformity of forming voltages was also more precise in the case of Au/Ti/HfAlO_x_/TiN-NP/HfAlO_x_/ITO RRAM devices due to the presence of ALD-based TiN-NP.

Also, during the first RESET, the improvement in uniform memory window as well as lower RESET voltages was observed, as shown in Fig. 2d. Similar improvement was monitored by Wu et al. in their study with nanocrystals-based RRAM devices [25]. Figure 2b and e is the typical bipolar resistive switching characteristics of both devices. In both devices, the SET and RESET properties were clearly demonstrated. After introducing TiN-NP, the memory window was clearly increased, as shown in Fig. 2e. In the supporting information (Additional file 1: Fig. S4), the comparison characteristics of bipolar switching characteristics, endurance properties of first 500 cycles, and the distribution of LRS and HRS have been given for both devices. In the Au/Ti/HfAlO_x_/ITO RRAM device, the conductive filaments (CFs) form in the canonical shape between top and bottom electrode during the electroforming process. The presence of tri-valent Al atoms inside tetra-valent HfO_2_ transition oxide creates intrinsic oxygen vacancies (V_O_). Also, it is already reported that the reactive Ti electrode can scavenge O-ions (formation of thin TiO_x_ layer) from underlying HfAlO_x_ insulator due to its weakly bonded O-atoms [26]. So in the SET process, V_O_ is accumulated near the CFs and the oxygen ions (O^‒^) are depleted due to the external electric field, which further connects the top and bottom electrode and brings the device into a low-resistance state (LRS) [27]. Schematic illustration is described in the inset of Fig. 2b. During RESET operation, the O^‒^ migrated back to the tip of the CFs and recombined with V_O,_ narrowing the CFs and increasing the device's resistance (HRS). As shown in Fig. 2e, the enhancement in the memory window can be the results from denser and bigger CFs due to the surface defects (originated from TiO_x_N_y_/HfAlO_x_ interface) of embedded TiN-NPs inside HfAlO_x_ [9]. The enhancement of I_on_/I_off_ ratio during the endurance test up to 1000 cycles of NP-embedded RRAM device clearly indicated that the induced oxygen vacancy generation was prominent due to the larger effective area of TiN-NP surface. Also, according to Gao et al., the presence of metal NPs inside switching insulators enhances the local electric field, which further helps the formation of CFs and rupture during the RESET process [16]. The inset of Fig. 2e shows the local field enhancement and formation and rupture of CFs during SET and RESET due to TiN-NPs present inside amorphous HfAlO_x_ matrix. A similar phenomenon has also been reported earlier by Liu et al. [14]. According to Gao et al. and Liu et al., Ag and Cu NPs inside the switching layers enhance the electric field around the NPs and form the conical shaped conducting filament, improving the uniformity of the witching cycles and reducing the switching electric field. However, these NPs may dissolve into Ag or Cu cations during the switching process. These mobile metal ions can migrate and deposit on the electrodes. So, the SET and RESET process involves both oxygen vacancy and migrated metal ions, as reported previously by Gao et al. and Liu et al. In this experiment, the TiN-NP does not dissolve after applying an external electric field and maintaining the original shape. Also, the surface oxidation of TiN-NP helps form a TiO_x_N_y_/HfAlO_x_ interface which further allows controlled stepwise gradual SET and RESET characteristics.

**Fig. 3:**
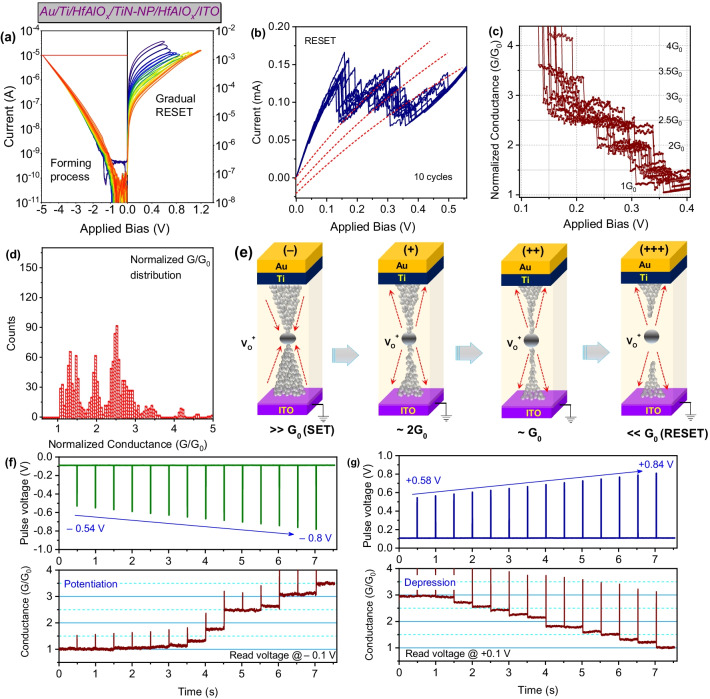
**a** Gradual electroforming and RESET process with stepwise increment of applied bias at both polarities shows multilevel memory properties. **b** Quantized conductance properties of TiN-NP-based RRAM device by I‒V characteristics during DC voltage sweep RESET characteristics. **c** Corresponding Normalized conductance (G/G_0_) vs voltage curves from multiple cycles. **d** Histogram obtained from the G/G_0_ collected from multiple cycles. **e** Schematics of the narrowing in conductive filaments during progressively increasing of RESET voltage. **f**, **g** Increasing and decreasing of quantized conductance with negative pulse from ‒0.54 V/1 ms to ‒0.0.8 V/1 ms, and positive pulse from + 0.58 V/1 ms to + 0.84 V/1 ms.

To evaluate the atomic point contacts (APCs) in Au/Ti/HfAlO_x_/TiN-NP/HfAlO_x_/ITO RRAM device with quantized conductance, the electroforming and first RESET of the device were restricted with minimum current compliance and RESET voltage as shown in Fig. 3a. CFs were found to be formed by slowly increasing the forming voltage with a step of ‒0.05 V, and at ~ ‒5.0 V, electroforming was observed at I_cc_ of 10µA. Similarly, during the first RESET of the device, with a step voltage of + 0.05 V, full RESET was achieved at + 1.2 V. By tuning the conductive filament size, the quantized conductance states were controlled due to APCs creation and annihilation process [4, 28]. Figure 3b shows the RESET I-V characteristics with the distinguishable stepwise decrease in currents, indicating different quantized conductance levels. With similar behavior, repetitive ten cycles were recorded in Au/Ti/HfAlOx/TiN-NP/HfAlOx/ITO RRAM device. As the voltage sweep rate is critical to achieving conductance quantization behavior, during the measurement, a very slow DC sweep rate was applied (0.002 V/step) as the change in quantized states lasted for a very narrow voltage region [29]. Normalized conductances (G/G_0_) have been plotted in Fig. 3c, which shows initially the conductance jumps down to ~ 3.5G_0_ due to CFs dissolution atom-by-atom. Continuously increasing the RESET step voltage, the conductance of the devices becomes stable at integer and half-integer multiples of ~ 2.5 G_0_, ~ 2 G_0_, and ~ 1.5 G_0,_ which clearly shows the quantized conductance behavior. Although the physical reason for the half-integer conductance is yet to be explored, according to Shu et al., the possible explanation is assumed to be due to the presence of diffused other atomic metal impurities near the CFs shift Fermi energy level of CF [30]. Similar half-integer conductance was also observed in ZnO, HfO_2_, Nb_2_O_5_, SiO_2_ oxide-based switching layers [4, 5, 19, 31].

The statistical distribution of the normalized quantized conductance is plotted in Fig. 3d. Conductance peaks were found to be concentrate near the integer, and half-integer multiples of G_0_ counted with quantized conductance states in every 0.1G_0_/step as discussed above. So this phenomenon reflects the fact that TiN-NP-based HfAlO_x_ switching layers are suitable for conductance quantization during the switching process. In this case, due to the presence of TiN-NP, the nanoscale CFs formation during forming process give an ideal condition to realize the quantized conductance [32]. Schematic illustration is drawn in Fig. 3e, which explains the CF formation and dissolution of CF by O^‒^ recombination to V_O_. The V_O_ and O^‒^ are separated during the forming process and form the localized CFs assisted by the TiN-NPs inside HfAlO_x_ at negative applied voltage to the top electrode. After successively increasing RESET voltage step by step by positive electric field at the top electrode, the conductance decreases driven by oxygen ions that recombine O^‒^ to V_O_ in an atomic scale. Precise control of quantized conductance state also has been demonstrated by both negative pulse from ‒0.54 V/1 ms to ‒0.8 V/1 ms during potentiation and positive pulse from + 0.58 V/1 ms to + 0.84 V/1 ms during depression at interval of 0.5 s as shown in Fig. 3f, and g. Increasing/decreasing conductance at the read voltage of ± 0.1 V as shown in Fig. 3 confirmed the change of conductance by increasing pulse amplitude. During application of negative pulse, initially, the normalized conductance ~ 1G_0,_ and after application of negative pulse amplitude up to ‒0.8 V, the conductance increases to ~ 3.5G_0_. A similar decrease in conductance was observed with stable integer and half-integer of G_0_ after applying pulse amplitude up to ‒0.84 V. This behavior shows the stability of the CFs due to the presence TiN-NP inside HfAlO_x_ matrix. Similar behavior of conductance control is also demonstrated by Younis et al. and Gao et al. [29, 33]. Details of measurement techniques and plotting for the quantized conductance are given in the supplementary material under Additional file 1: Fig. S6(a), and (b).

**Fig. 4:**
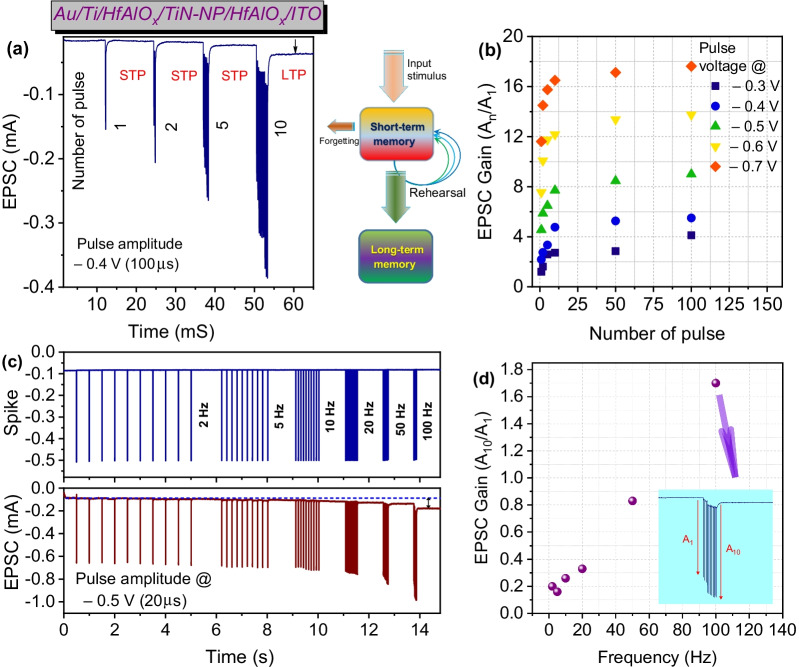
**a** Transition from STM to LTM of Au/Ti/HfAlO_x_/TiN-NP/HfAlO_x_/ITO RRAM device with the change of EPSC varying the spike number from 1 to 10 at the pulse voltage amplitude of ‒0.4 V/100 µs, along with the schematic of memory enhancement by repeated pulses. **b** EPSC gain (A_n_/A_1_) plotted w.r.t. the pulse number at different pulse voltage from ‒0.3 V/100 µs to ‒0.7/100 µs V. **c** Synaptic plasticity EPSC measured with frequency from 2 to 100 Hz and **d** the EPSC gain (A_10_/A_1_) at pulse amplitude of ‒0.5 V/20 µs.

To emulate the biological synapse through the synaptic weight changes is known as synaptic plasticity which includes short-term plasticity (STP) and long-term potentiation (LTP) and can be measured by excitatory postsynaptic current (EPSC) [34–36]. Also, the conversion from STP to LTP process in biological synapses can be possible by rehearsing the applied stimulation [37]. Synaptic weight (SW) change has been recorded to evaluate these characteristics depending on the increasing spike number and frequency, as shown in Fig. 4a–d. The EPSC was found to be increased gradually depending on the spiking number, as shown in Fig. 4a. In supporting information, increasing SW has been presented under different applied pulse voltage amplitude (at ‒0.3 V/100 µs to ‒0.7 V/100 µs) with pulse number of 1 to 10 as shown in Additional file 1: Fig. S5. It was clearly demonstrated as expected depending on the pulse amplitude and numbers, EPSC increased gradually in accordance with the presynaptic spike. Similar EPSC increment behavior dependence of presynaptic spikes voltage was observed by Yu et al*.* [38]. Maximum EPSC recorded during the first and final pulse input was denoted by the A_n_ and A_1_. EPSC gain (A_n_/A1) plotted in Fig. 4b clearly demonstrates the SW increment depending on the pulse number and spike amplitudes. Short-term memory (STM) to long-term memory (LTM) transition was observed after increasing the spike number from 1(STM) to 10(LTM) at the spike voltage of ‒0.4 V/100 µs, as the postsynaptic current at the base voltage of ‒0.1 V increased (resting current) indicated by the arrow in Fig. 4a. Similar transition behavior has been observed by Kim et al. and Chen et al. [36, 39]. It is interesting to note that at low spike voltage (‒0.3 V/100 µs), even after the application of 10 pulses, memory transition did not occur at the read voltage of ‒0.1 V (shown in Additional file 1: Fig. S5), which confirms the short-term plasticity due to weak filament formation [35]. Depending on the repetition of spikes, the STM to LTM transition increased the filament size accumulating the O-vacancies near the TiN-NPs surface inside the HfAlO_x_ matrix. The schematic model transition from STP to LTP depends on the process of rehearsal repetition, as shown in Fig. 4a [36]. After the application of 1 to 5 pulse stimuli, the conductance temporarily increases, but due to the weak filament formation, the synaptic weight quickly returns to its initial state. Although after application of 10 and more stimuli at the same spike amplitude, highly increased conductance of synapses was observed due to induced strong filaments formed by the O-vacancies. As a result, the CFs found not to be ruptured quickly, and consequently, the pulse-induced O-vacancy inside the switching layer remained stored, leading to LTM.

Absorption of O-vacancy near the conductive filaments can also be controlled by altering the spike frequency. Synaptic filtering characteristics (spike rate-dependent plasticity, SRDP) were also studied with increasing applied pulse frequency from 2 to 100 Hz (decreasing the spike interval) [40]. As shown in Fig. 4c, the pulse amplitude was constant at ‒0.5 V/20 µs and the representative EPSC was presented by applying 10 consecutive presynaptic pulses with different frequencies. The EPSC gain (A_10_/A_1_), as shown in Fig. 4d, was increased from 0.2 to 1.7 by increasing the frequency from 2 to 100 Hz confirmed the filtering characteristics. At low frequency (with longer delay time), the EPSC changes due to the applied spike but due to the longer delay, most of the accumulated O-vacancies migrated back, leading to less EPSC gain[34, 41–43]. Although due to the higher frequency enhanced EPSC gain achieved, the diffused O-ions (formation of O-vacancy) were unable to reach back to its initial state with such a short time[34]. As shown in Fig. 4c, the arrow on the resting current after 100 Hz pulse frequency indicates that the STM turns into LTM with stronger intensity. A magnified EPSC at the pulse frequency of 100 Hz is presented in the inset of Fig. 4d. So, TiN-NP inserted HfAlO_x_ switching layer properties can mimic synaptic dynamic high-pass filtering characteristics, which is very important for neuromorphic computing [44]. The measurement details and data processing method for EPSC variation under different spike number and frequency scheme are described in Additional file 1: Fig. S7 and Fig. S8.

## Conclusion

In summary, we have demonstrated a well-controlled stepwise change of quantum conductance in the Au/Ti/HfAlO_x_/TiN-NP/HfAlO_x_/ITO RRAM device. Atomic layer deposition of TiN-NP enables controlling a very low thickness of the overall switching layer. The presence of TiN-NP inside the HfAlO_x_ matrix enhances the memory window due to the TiO_x_N_y_/HfAlO_x_ interface on the surface of TiN-NP, which helps for gradual change in conductance in atomic scale. The conductance quantization and multilevel memory behaviors controlled by TiN-NP inside HfAlOx show the suitability for implementing high-density memory storage. A conductance state transition from short-term plasticity to long-term potentiation behavior also suggests that HfAlO_x_/TiN-NP/HfAlO_x_ switching layer can be mimicked similar to biological synapses.

## Supplementary Information

**Additional file 1.** Supporting information.

## Data Availability

All data generated or analyzed during this study are included in this article and its supplementary information file.

## Abbreviations

NP: Nanoparticles
RRAM: Resistive random access memory
ALD: Atomic layer deposition
TiN: Titanium nitride
CF: Conductive filament
DC: Direct current
NCs: Nanocrystals
APCs: Atomic point contacts
STP: Short-term plasticity
LTP: Long-term potentiation
ITO: Indium tin oxide
TEs: Top electrodes
HRS: High-resistance states
LRS: Low-resistance states
FIB: Focused ion beam
TEM: Transmission electron microscope
RS: Resistive switching
EDS: Energy-dispersive spectroscopy
SEM: Scanning electron microscopic
STEM: Scanning transmission electron microscopy
EPSC: Excitatory postsynaptic current
SW: Synaptic weight
