# Supplementary material for: Synaptic Plasticity and Quantized Conductance States in TiN-Nanoparticles-Based Memristor for Neuromorphic System
Source: Nanoscale Res Lett. 2022 Jun 10;17:58. doi: 10.1186/s11671-022-03696-2 (PMC9187820; doi:10.1186/s11671-022-03696-2)
Supplement: Supplementary file 1 — Additional file 1. Supporting information. [file 11671_2022_3696_MOESM1_ESM.docx]

**Supplementary materials:**

**Synaptic plasticity and Quantized Conductance States in TiN-nanoparticles based memristor for neuromorphic system**

Chandreswar Mahata^a^, Muhammad Ismail^a^, Myounggon Kang^b*^, Sungjun Kim^a*^

^a^Division of Electronics and Electrical Engineering, Dongguk University, Seoul 04620, Republic of Korea

^b^Department of Electronics Engineering, Korea National University of Transportation, Chungju-si 27469, Republic of Korea

* Corresponding author: [mgkang@ut.ac.kr](mailto:mgkang@ut.ac.kr) (M. Kang) and [sungjun@dongguk.edu](mailto:sungjun@dongguk.edu) (S. Kim)


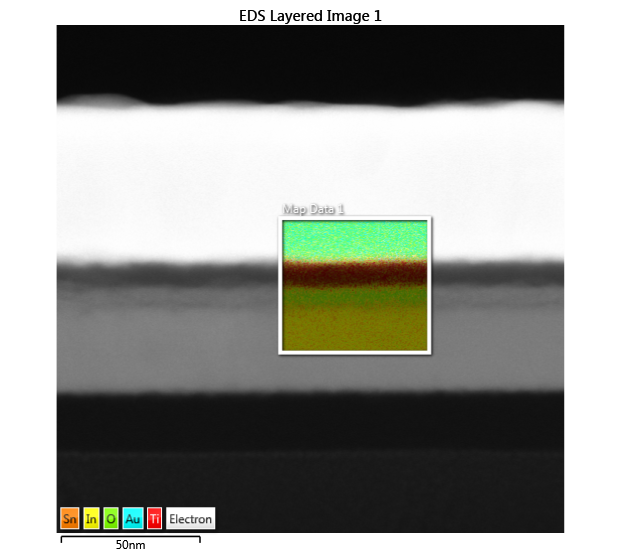


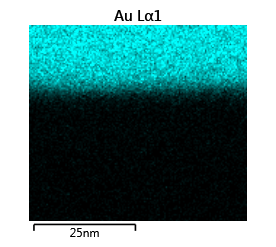

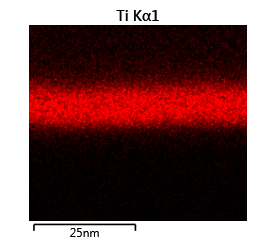


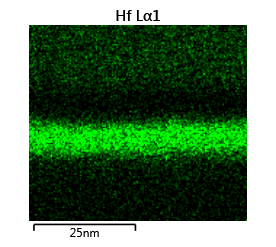

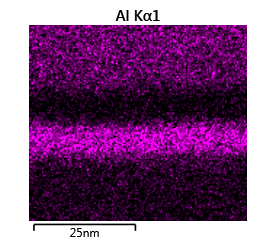


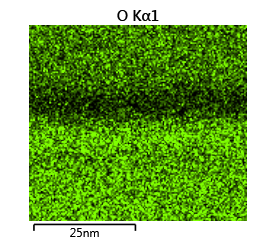

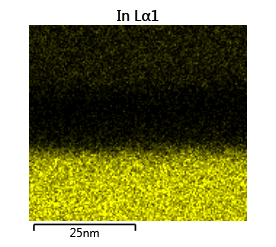


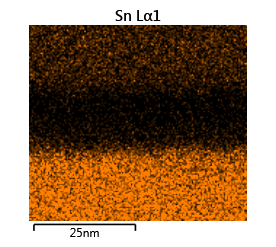


**Figure S1**: Cross-sectional STEM image of Au/Ti/HfAlO_x_/TiN-NP/HfAlO_x_/ITO and EDS elemental mapping of Au, Ti, Hf, Al, O, In and Sn.


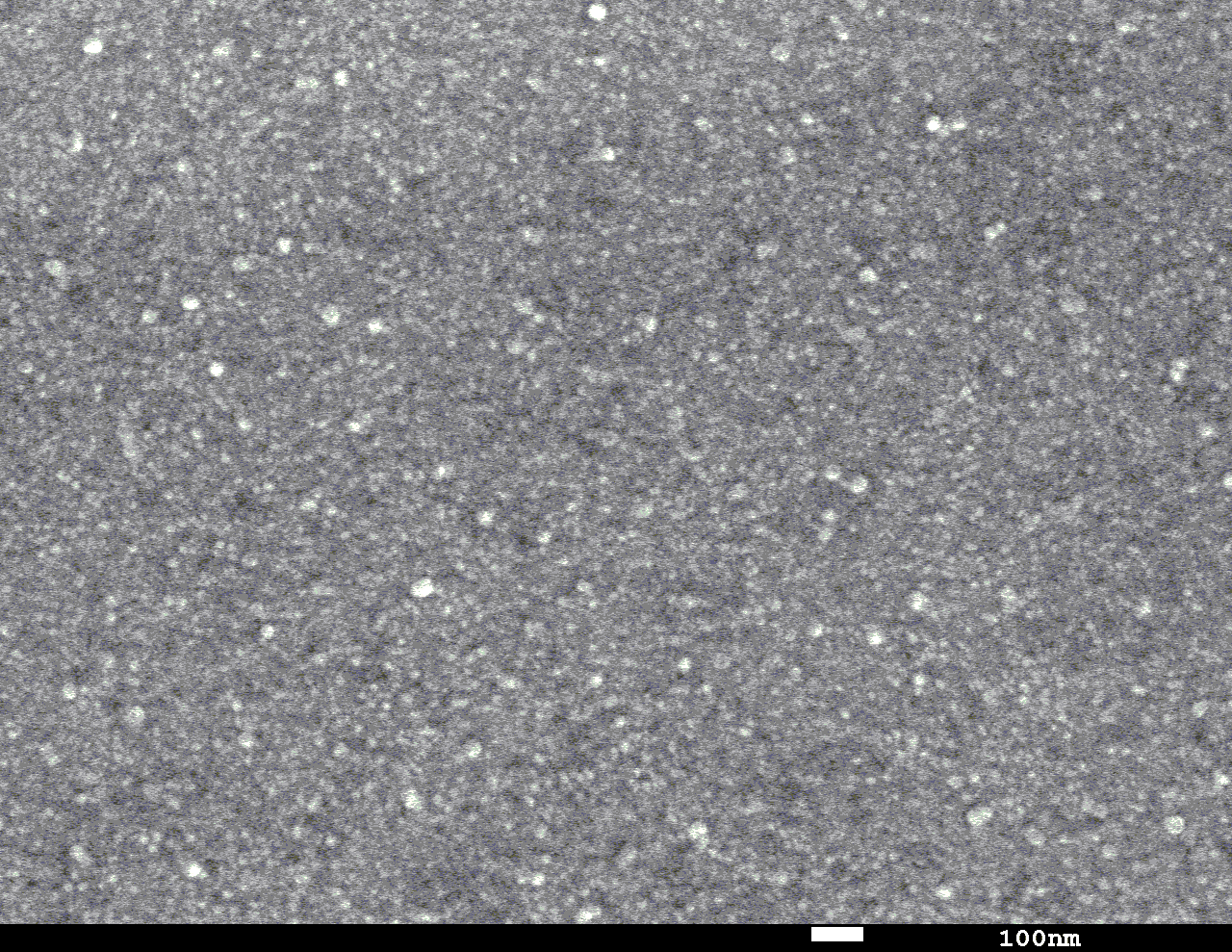


Au

Ti

HfAlO_x_

TiN-NP

ITO

~2 nm

~5 nm

~25 nm

~10 nm

**Figure S2:** Large area SEM image of atomic layer deposited TiN-NP on the HfAlO_x_ dielectric film and schematic diagram of different diameter of the NPs were observed by SEM.

**Figure S3:** Comparison of electroforming electric field at 10 µA I_cc_ multiple Au/Ti/HfAlO_x_/ITO and Au/Ti/HfAlO_x_/TiN-NP/HfAlO_x_/ITO RRAM devices.

(a)

(b)

(c)

Figure S4: (a) Comparison of SET/REST properties, (b) first 500 cycles of endurance characteristics, and (c) distribution of LRS and HRS, for Au/Ti/HfAlO_x_/ITO and Au/Ti/HfAlO_x_/TiN-NP/HfAlO_x_/ITO RRAM devices.

**Figure S5:** Applied pulse sequence with number of pulse from 1 to 10. Ten cycles of excitatory postsynaptic current (EPSC) recorded at different pulse voltage from ‒0.3 V/100 µs to ‒0.7/100 µs V varying the spike number from 1 to 10.


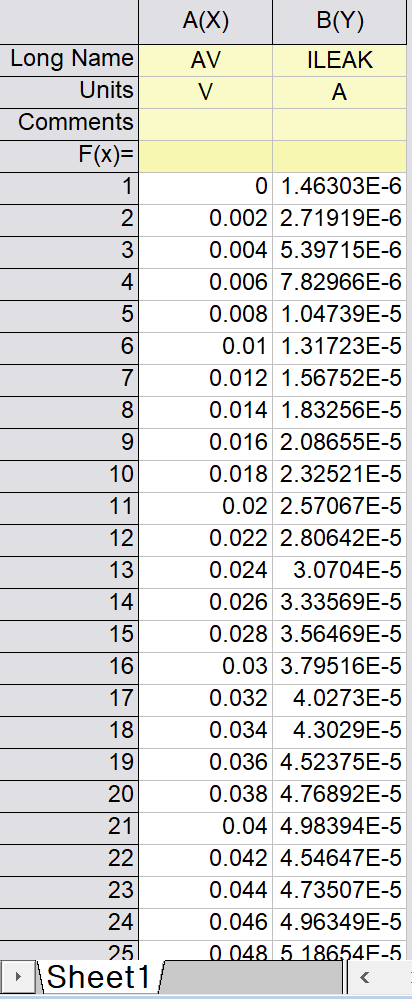


(a)

(b)

**Figure S6**: Data processing method for plotting the quantized conductance behavior with (a) the DC voltage sweep measurement, under a very slow sweep rate (0.002 V/step), (b) Increasing of quantized conductance with negative pulse from ‒0.54 V/1ms to ‒0.0.8 V/1ms.

**Figure S7:** Data processing method for calculating the change of EPSC varying with the spike number from 1 to 100 at the pulse voltage amplitude of ‒0.4 V/100 µs.

**Figure S8:** Data processing method for calculating the change of EPSC varying with the spike frequency from 2 to 100 Hz at the pulse voltage amplitude of ‒0.5 V/20 µs.

The quantized conductance state was achieved by a very slow voltage sweep rate (0.002 V/step) during the measurement, as the change in quantized states lasted for a very narrow voltage region. In Fig. S6(a) we have given the data set and the I-V curve obtained for one cycle of DC RESET characteristics. In the next step the conductances were calculated by the Eqn. G=I/V (S, siemens = amps per volt). The normalized conductance was achieved dividing the obtained G by G_0_. The normalized quantized conductance distribution has been plotted using the Histogram function in the OriginLab graphing software.

Similarly, the quantized conductance states were obtained during potentiation and depression applying pulse voltage. For simplicity in Fig. S6(b), we described the quantized conductance state achieved during potentiation. The pulse voltage and EPSC were plotted together at the top. At the base voltage or the read voltage, the current change by increasing pulse amplitude was clearly shown (Magenta color). The normalized conductances were calculated and plotted below in Fig. S6(b) following the method as mentioned above.

In Fig. S7, and S8, the increment of EPSC was found to be increased depending on the pulse number and pulse frequency as shown below. Irrespective of the pulse amplitude, the EPSC (the peak current at applied pulse) continuously increased up to the application of 10 spikes. After that the EPSC was somehow saturated at the pulse number of 50 to 100, which is clearly shown in Fig. S7. EPSC gain (A_n_/A1) is plotted and indicated by different colors due to different pulse numbers in Fig. S7. As described in Fig. S8, a similar approach has been followed to calculate the EPSC gain with increasing applied pulse frequency from 2 Hz to 100 Hz. Although in this measurement, the number of spikes was always kept constant at each frequency (10 numbers). The EPSC gain (A_10_/A_1_) was calculated from the current ratio obtained from the 10th pulse to the 1st pulse in each frequency.
